# Contribution of a multicomponent intervention on functional capacity and independence on activities of daily living in individuals with neurocognitive disorder

**DOI:** 10.1186/s12877-021-02591-2

**Published:** 2021-11-03

**Authors:** Flávia Borges-Machado, Duarte Barros, Laetitia Teixeira, Oscar Ribeiro, Joana Carvalho

**Affiliations:** 1 CIAFEL - Research Centre in Physical Activity, Health and Leisure, Faculty of Sports, University of Porto, Rua Dr. Plácido Costa 91, 4200-450 Porto, Portugal; 2 Faculty of Sports, University of Porto, Porto, Portugal; 3 ITR – Laboratory for Integrative and Translational Research in Population Health, Porto, Portugal; 4 School of Medicine and Biomedical Sciences, University of Porto, Porto, Portugal; 5 CINTESIS - Center for Health Technology and Services Research, Porto, Aveiro, Portugal; 6 Department of Education and Psychology, University of Aveiro, Aveiro, Portugal

**Keywords:** Dementia, Physical activity, Functionality, Physical fitness

## Abstract

**Background:**

To examine the effects of a 6-month multicomponent (MT) exercise intervention in the functional capacity and ability to independently perform activities of daily living (ADL) of individuals diagnosed with neurocognitive disorder (NCD).

**Methods:**

A quasi-experimental controlled trial with a parallel design study was conducted in multicentered community-based settings. Forty-three individuals (N Female: 30) were allocated to an exercise group (EG; N: 23; mean 75.09, SD = 5.54 years) or a control group (CG; N:20; mean 81.90, SD = 1.33 years). The EG engaged in a 6-month MT program (60-min sessions, twice a week). Exercise sessions were divided into a warm-up, specific training (e.g., coordination and balance, lower and upper body strength, and aerobics), and cool down. Lower body function, mobility, and gait speed were evaluated through Short Physical Performance Battery (SPPB), Timed-Up and Go test (TUG) and 6-Meter Walk test, respectively. The Barthel Index (BI) was administered to assess individuals’ ADL independence. Evaluations were performed before and after the 6-month intervention.

**Results:**

Linear Mixed Models revealed a statistically significant interaction (time X group) effect factor on SPPB (B = 2.33, 95% CI: 1.39–3.28, *p* < 0.001), TUG (B = − 11.15, 95% CI: − 17.23 – − 5.06, *p* = 0.001), and 6-Meter Walk test (B = 0.17, 95% CI: 0.08–0.25, p < 0.001). No differences between groups or assessment moments were found in the ability of individuals to independently perform ADL.

**Conclusions:**

The 6-month MT exercise intervention improves the functional capacity of older adults living with NCD.

**Trial registration:**

ClinicalTrials.gov – identifier number NCT04095962; retrospectively registered on 19 September 2019.

## Background

Major neurocognitive disorder (NCD), often referred as dementia, can be diagnosed according to DSM-5 in the presence of four criteria, being acquired decline in one or more cognitive domains the core feature of this condition, which interferes with independence in daily activities [1]. Worldwide, major NCD is considered the greatest challenge of this century, as it is estimated to affect more than 152 million people by 2050 [2]. Particularly in Europe, the number of cases is expected to almost double from 9.8 to 18.8 million [3].

The understanding of major NCD etiology is still shifting. Currently, there are 12 potentially modifiable risk factors (e.g., hypertension, hearing impairment, obesity, depression, and physical inactivity) capable to prevent or delay up to 40% of all cases [2, 4]. Although aging is the greatest risk factor for all-cause major and minor NCDs, addressing physical inactivity should be prioritized, particularly considering the underlying direct neurological effects (e.g., increased neurogenesis, cerebral blood flow, and BDNF concentrations) and indirect influence on other modifiable cardiovascular risk factors of physical activity [5].

In this sense, after major NCD diagnosis, physical activity and specifically physical exercise are particularly relevant on counteracting/managing disease-related factors and associated symptoms [6, 7]. People living with major NCD exhibit increased dependency on activities of daily living (ADL) as this syndrome progresses [1]. Furthermore, the decline on basic activities such as feeding, bathing, and dressing, leads to decreased autonomy [8, 9] that may also be exacerbated by age-related physical function limitations. Indeed, community-dwelling older adults living with minor NCD and early major NCD tend to present decreased physical fitness when compared to their healthy-peers, particularly in lower-body strength, upper-and-lower body flexibility, agility/dynamic balance, and cardiorespiratory fitness [10], which are highly associated with one’s ability to perform daily activities [11]. Apart from these deficits in physical fitness abilities, they tend to exhibit reduced walking speed, gait disturbances [12, 13], impaired balance, and movement coordination [14].

Although the performance in ADL relies on both cognitive and physical abilities and their interaction with the living environment [15], increasing evidence from review studies and meta-analysis supports the beneficial role of regular exercise in the ability to independently perform ADL among individuals diagnosed with major NCD [16–18]. However, the appropriate training methodology, frequency, intensity, and duration of exercise sessions, program length, and outcome measurements are still to be determined in this specific population.

Multicomponent exercise interventions, combining muscle strengthening, aerobic resistance, and balance training [19] were recently recommended by the American College of Sports Medicine [11] and by the World Health Organization (WHO) [20] for individuals aged 65 years and older to improve functional capacity and prevent falls. The effectiveness of this training methodology has also been proved in older adults diagnosed with major NCD [21–24] but further evidence is needed to examine how multicomponent interventions may have an impact on functional capacity and physical fitness [25].

Functional capacity corresponds to “the composite of all the physical and mental capacities that an individual can draw on” [26], reflecting one’s ability to undertake various ADL without assistance, which in turn is directly associated with physical functioning. Given the association between functional capacity and quality of life of older adults with neurocognitive disorder [27], the need to minimize caregiver’s burden and prevent or delay institutionalization [28], it is urgent to determine the effectiveness of multicomponent training (MT) in improving physical abilities and daily functionality of individuals with NCD. As highlighted by the WHO [20], it is expected that this training methodology may have a positive impact on functional capacity of individuals with NCD, which in turn, can be reflected on their ability to independently perform ADL.

Therefore, this study aims to examine the contribution of a 6-month MT exercise intervention on the functional capacity (e.g., lower extremity function, mobility, and gait speed) in older adults living with NCD, and on their ability to independently perform ADL.

## Methods

### Study design and setting

The present study takes part in the “Body & Brain” project. This project is registered at the US National Institutes of Health clinical trials registry - ClinicalTrials.gov (ID: NCT04095962) and its study protocol can be found elsewhere [29]. A two-arm quasi-experimental controlled trial with a parallel design was conducted in multicentered settings in the metropolitan area of Porto city – Portugal, between September 2018 and May 2019.

This investigation was conducted in full compliance with the Declaration of Helsinki, and the Ethical Committee approval has been granted from the Institutional Review Board of the Faculty of Sports, University of Porto (Ref CEFADE22.2018). Following the best ethical procedures, informed consent was obtained from interested individuals with NCD and their legal representatives or significant person (complying with the legal requirements associated).

One hundred participants were recruited from public and private institutions (e.g., daycare centers, local community centers, and nursing homes), hospital centers and clinics (i.e., outpatients accompanied by psychiatrists or neurologists), Alzheimer’s and caregivers’ associations, municipalities, local journals, and social media.

**Fig. 1:**
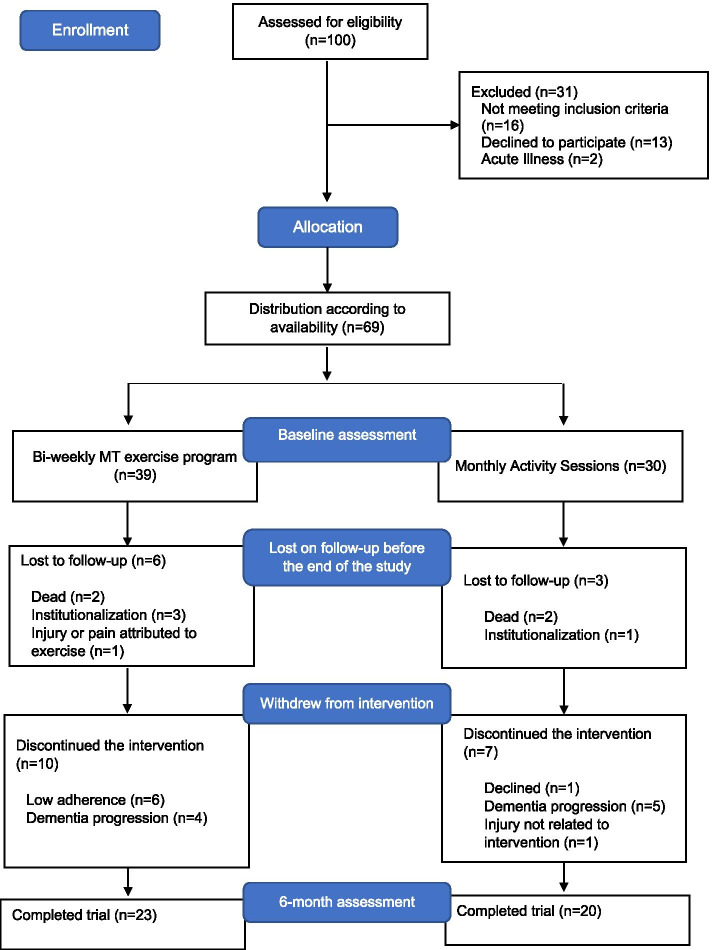
Study Flow Diagram

Eligible participants and their caregivers/legal representatives received a complete explanation of the study purposes, risks, and procedures. After agreeing to participate, individuals were allocated into a 6-month intervention – experimental group (EG), or to a social activity group – control group (CG), according to their availability to join one of the groups (Fig. 1). Therefore, both options were given to eligible participants who decided which group they wanted to adhere.

### Participants eligibility criteria

The eligible subjects pool was restricted to individuals with the following characteristics: i) age ≥ 60 years; ii) clinically diagnosed by a physician for at least 6 months with major neurocognitive disorder according to *Diagnostic and Statistical Manual of Mental Disorders* (DSM-IV-TR or DSM-5) [1], or with dementia according to the ICD-10 [30], or the NINCDS-ADRDA [31]; iii) capacity to walk autonomously without an assistive device or human assistance.

Individuals were excluded if the following criteria were present: i) diagnosed with any condition or disorder in which exercise is contraindicated, such as unstable or ongoing cardiovascular and/or respiratory and/or musculoskeletal condition; ii) recent hospitalization (e.g., previous month) and/or in recovering from surgery or rehabilitation; iii) presenting an advanced stage of major NCD (e.g., ≤ 10 points on MMSE) that could affect physical performance in the exercise training sessions or testing procedures.

### Interventions

#### Experimental group: MT intervention

MT program was implemented by professionals specialized in exercise, that prior to the program implementation received specific training concerning NCD clinical features and progression, challenging behaviors, communication strategies, and safety issues [32].

An adaptation period was implemented before the main MT training to promote familiarization with exercises and the learning of movements execution at a low intensity. This period was also determinant for participants’ socialization [33].

The exercise sessions took about 60-min each, were conducted twice a week for 6-months, and involved 5 to 8 participants; they were conducted during the late morning and/or early afternoon periods [32]. Routine, simple, and functional exercises were preferred. The intensity was monitored using heart rate monitors.

The exercise sessions were divided into 3 main parts, following the main guidelines recommended by the American College of Sports Medicine [11] and the World Health Organization [20]: warm-up (10 min, including slow walk, postural and mobility exercises for general activation, and stretching exercises); specific training (35–45 min, including balance/coordination training, strength, and aerobic exercises); and cool down (5 min with breathing and stretching exercises). After the warm-up, some coordination and balance exercises were included, by reducing the base of support and/or sensory input, and dynamic movements to disturb the center of gravity. Strength training included 4 to 6 multi-joint exercises involving major muscle groups. The number of repetitions decreased with the increasing load that could be lifted correctly to volitional fatigue. A rest period of 1′30’-2″ between the two sets was completed. Finally, aerobic endurance was implemented from two periods of 5-min (60–65% of HRmax) to two periods of 10-min of continuous exercise (75–80% of HRmax). Detailed information on the exercise protocol can be found elsewhere [29].

#### Control group: social activity

Participants in the control group received monthly sessions regarding physical activity and information on health-related topics as a complement to standard care. No specific exercise intervention was conducted with this group. Participants were contacted regularly via phone calls to ensure retention and motivation.

### Data collection

Data collection occurred at baseline, and after the 6-months intervention. Evaluations were conducted by trained researchers, who were not masked to group assignment and were not responsible to implement the exercise program. The adherence rate was calculated considering the total number of attended sessions through the total offered. A cutoff of 70% adherence was considered.

### Outcome measures

#### Sociodemographic characteristics and general clinical data

Sociodemographic information such as age, gender, marital status, education, and living situation was gathered via a screening questionnaire with caregivers / legal representatives. Pharmacological treatment of participants (total number and type of prescribed daily medications) was also collected. A scale of relevant comorbid conditions (ranging from 0 to 15) was created to gather information and characterize the general health of individuals; it comprised the following conditions: diabetes, dyslipidemia, hypertension, myocardial infarction, cardiac insufficiency, peripheral vascular disease, stroke/transient ischemic attack, other cardiovascular diseases, osteoporosis, arthrosis, rheumatoid arthritis, respiratory disease, renal disease, or other diseases (e.g., history of cancer).

#### Lower body function

The Short Physical Performance Battery (SPPB) [34] was used to assess lower extremity function; it evaluates static balance, gait ability, and lower limb strength. The final score is the sum of points from each of the three subtests, ranging from zero (worst performance) to 12 points (best performance). A 1-point change in the total score has clinical relevance [35]. The SPPB has shown excellent test-retest reliability, predictive and convergent validity [36], and good internal consistency (Cronbach’s α = 0.76) [34]. Moreover, the SPPB has shown to be a valid instrument to screen frailty and predict disability, institutionalization, and all-cause mortality [37].

#### Mobility

The Timed-Up and Go test (TUG) [38] is a gold-standard test to evaluate older adults’ functional mobility and has been widely used in individuals living with major NCD [39]. This test does not require special equipment or training; it is simple, rapid to apply, and sensitive to identify those who are at increased risk for falls [40], and has been considered a predictive marker of functional dependency occurrence [41]. Participants were requested to rise from a standard armchair, walk at a normal pace a 3-m distance turning around the ground mark positioned in front, return, and sit down again. Time started after the word “go” sounded and ended once the participant sat back down on the chair. The lowest time (indicative of better performance) of the two trials was considered. Excellent relative test-retest reliability has been found on the TUG (ICC ≥ 0.94) [42, 43] in older adults with major NCD, from mild to severe stages.

#### Gait speed

The 6-Meter Walk test [43] was used to evaluate participants’ gait speed. Individuals were asked to walk in a 6-m straight line at their normal pace. The time was registered for each of the two allowed attempts, and the best value, converted to walking speed (m/s), was considered. Gait speed was recognized as a reliable outcome measure to use with people diagnosed with major NCD [42] and has been extensively used with this population [14, 24, 43].

#### Daily functionality

The Barthel Index (BI) [44] was used to assess individuals’ ability to independently perform ADL, and was applied to the caregiver/ legal representative/ significant person. With a total score ranging from zero to 100, this questionnaire addresses ten basic daily activities: feeding, bathing, grooming, dressing, using the toilet, continence of bowels and bladder, transfers from bed to chair and return, and ambulation (on surfaces levels and climbing stairs). Lower scores are indicative of higher dependency levels. The Portuguese version of BI [45] has shown a good internal consistency (Cronbach’s α = 0.89).

### Statistical analyses

Data normality was verified using Shapiro-Wilk test. Measures of central tendency and dispersion, presented as mean and standard deviation (SD), or median and interquartile range, and frequencies (percentages) were used as appropriate to describe sample characteristics. Between groups comparisons at baseline were performed with both parametric (independent t-test) and non-parametric approaches (Mann-Whitney U test) for continuous variables, and Pearson’s Chi-Squared test or Fisher’s Exact test for categorical variables. Longitudinal changes in the outcome measures from baseline to 6-months were analyzed using linear mixed-effects models, considering group, time, and interaction time*group as fixed effects and participant as random effect. Adjustments were performed for age and gender. The least square mean within-group difference was estimated from these models. All statistical procedures were carried out with SPSS IBM Statistical version 26.0 or with R software version 4.0.4. A significance level of *p = 0.05* was established.

## Results

### Participants

Sixty-nine individuals diagnosed with NCD (mean 79.50 years old (SD = 6.73), 72.5% female) were included in the “Body & Brain” project and distributed between the EG and CG according to their availability and preferences to adhere one of the groups. From those, 26 participants were lost to follow-up (*n* = 9) or discontinued the intervention (*n* = 17), which translates into a dropout rate of 38%. The flow-chart of participants during the trial is shown in Fig. 1. Forty-three older adults (mean 78.26 years old (SD = 6.68), 69.8% female) have completed the intervention, 23 in the EG and 20 in the CG. Within both groups, major NCD diagnosis were established for at least 6 months and a maximum of 12 years. Twenty-seven individuals (62.8%) had an unspecified diagnosis of major NCD or dementia, 8 (18.6%) due to Alzheimer’s disease, 3 (7.0%) due to multiple etiologies, 3 (7.0%) due to Parkinson’s disease, 1 (2.3%) due to Lewy bodies disease, and 1 (2.3%) due to Korsakoff syndrome.

Study population baseline characteristics did not significantly differ between the experimental and control groups, except for age and number of diagnosed chronic comorbidities (Table 1). Dyslipidemia (74.4%), hypertension (69.8%), and diabetes mellitus (32.6%) were the most prevalent diseases in both groups. At baseline, our study sample presented a mean value on BMI indicative of overweight (mean 28.87 kg/m^2^ (SD = 4.88)), and 40% (*n* = 16) reported the occurrence of falls (at least one) over the period of 12-months prior to the baseline evaluation moment. Seventeen (73.9%) of the participants from the EG were female, the majority were married (52.2%) and had at least 4 years of formal education (69.6%). Cognitive function measured with both MMSE and ADAS-Cog was not statistically different between groups.

**Table 1:** Baseline sample characteristics

Characteristics	Total (***n*** = 43)	EG (***n*** = 23)	CG (***n*** = 20)	Statistical Inference
Age (years), mean (SD)	78.26 (6.68)	75.09 (5.65)	81.90 (1.33)	*p* < 0.001 ^a^
Age, range	61–89	61–83	70–89	
Gender (female), n (%)	30 (69.8%)	17 (73.9%)	13 (65%)	*p* = 0.526 ^b^
Civil Status, n (%)				
Widow	22 (51.1%)	10 (43.5%)	12 (60%)	*p* = 0.395 ^c^
Married or Civil Union	18 (41.9%)	12 (52.2%)	6 (30%)	
Divorced or Separated	3 (7.0%)	1 (4.3%)	2 (10%)	
BMI (kg/m^2^), mean (SD)	28.87 (4.88)	29.72 (5.39)	27.98 (4.23)	*p* = 0.257 ^a^
Year of Formal Education, mean (SD)	4.00 [3.00–8.25]	4.00 [3.00–9.00]	4.00 [3.00–6.00]	*p* = 0.676 ^d^
Education levels, n (%)				
Low (1–3 years)	13 (31.0%)	7 (30.4%)	6 (31.6%)	*p* = 0.858 ^c^
Medium (4–6 years)	18 (42.8%)	9 (39.2%)	9 (47.4%)	
High (7–12 years)	11 (26.2%)	7 (30.4%)	4 (21.0%)	
Number of Medications, mean (SD)	7.00 [5.00–9.00]	8.00 [5.00–10.00]	7.00 [5.00–9.00]	*p* = 0.571 ^d^
Number of Comorbidities, mean (SD)	4.02 (2.06)	4.61 (1.94)	3.35 (2.03)	*p* = 0.045 ^a^
Fallers in past 12 months, n (%)	16 (40%)	9 (40.9%)	7 (38.9%)	*p* = 0.897 ^b^
MMSE, mean (SD)	20.93 (4.84)	20.74 (5.10)	21.15 (4.64)	*p* = 0.785 ^a^
ADAS-Cog, mean (SD)	30.29 (11.68)	30.05 (12.15)	30.55 (11.44)	*p* = 0.891 ^a^

*Notes*: Values are presented as mean (SD) (continuous variables), median [interquartile range] or percentage (categorical variables)

*ADAS-Cog* Alzheimer’s Disease Assessment Scale – Cognitive *BMI* Body Mass Index *MMSE* Mini Mental State Examination

^a^: t – Independent sample T-test; ^b^: χ^2^ – Pearson’s Chi-Square test; ^c^: Fisher’s Exact test; ^d^: U – Mann-Whitney U test

Missing: 1 – Education levels, years of formal education and ADAS-Cog, 2 – BMI, 3 – Fallers in the past 12 months, 8 – Years after diagnosis

Over the intervention period, adherence to the bi-weekly exercise sessions was higher than 75% (mean 87%), considering the total number of sessions within the 6 months. No serious adverse events occurred during this intervention.

### Outcomes

After a 6-month multicomponent exercise intervention, the EG improved their functional capacity on SPPB, TUG, and 6-Meter Walk test. As opposite, the CG performance on these outcome measurements worsened.

Table 2 shown least square mean scores of SPPB and 6-Meter Walk test according to group, and they increased over time on EG, indicative of better functional capacity, and decreased in CG. The time spent raising up from a chair, turn around the ground marker and return to a seated position increased in individuals in the CG, as opposed to those in the EG. As described on Table 3, results from unadjusted fixed effects models revealed an interaction (time X group) effect factor on lower body function, mobility and gait speed measured through SPPB (B = 2.37, 95% CI:1.43–3.32, *p* < 0.001), TUG (B = − 11.38, 95% CI: − 17.48 – − 5.29, *p* = 0.001), and 6-Meter Walk test (B = 0.17, 95% CI: 0.08–0.25, *p* < 0.001), respectively. These results confirmed that changes on SPPB, TUG and 6-Meter Walk test across time were different according to the group.

**Table 2:** Within-group differences from baseline in the SPPB, TUG, 6-Meter Walk test and BI

		Experimental		Control	
		***n***	Mean (SE)^**a**^	***n***	Mean (SE)^**a**^
SPPB	M1	23	7.89 (0.48)	20	8.04 (0.51)
	M2	22	8.95 (0.52)	20	6.78 (0.53)
TUG	M1	23	15.7 (2.88)	20	14.8 (2.41)
	M2	22	11.8 (2.55)	19	22.0 (2.62)
6-Meter Walk test	M1	23	0.75 (0.04)	20	0.699 (0.04)
	M2	22	0.88 (0.04)	19	0.665 (0.05)
BI	M1	23	85.7 (2.90)	23	81.6 (3.10)
	M2	19	86.1 (3.09)	20	81.5 (3.27)

*a* Least square mean calculated from linear-effects mixed models, averaged for gender

*SE* standard error*, SPPB* Short Physical Performance Battery, *TUG* Timed-Up and Go Test, *BI* Barthel Index, M1 Baseline, M2 After 6-month Intervention

Significant difference within-group from baseline (*p* < 0.05)

**Table 3:** Unadjusted linear mixed models for SPPB, TUG, 6-Meter Walk test and BI

	Fixed Effects								
	Time			Group			Time X Group		
	B	95% CI	***p value***	B	95% CI	***p value***	B	95% CI	***p value***
SPPB	−1.28	− 1.96 – − 0.60	< 0.001^a^	1.08	−0.33 – 2.48	0.138	2.37	1.43–3.32	< 0.001^c^
TUG	7.24	2.74–11.68	0.003^a^	− 1.80	−7.76 – 4.16	0.558	−11.38	− 17.48 – −5.29	0.001^c^
6-Meter Walk test	−0.03	− 0.10 – 0.03	0.210	0.11	0.003–0.22	0.049^b^	0.17	0.08–0.25	< 0.001^c^
BI	−0.08	−4.81 – 4.62	0.973	7.57	−0.16 – 15.29	0.060	0.67	−5.51 – 6.84	0.833

Group: control group is reference. Time: baseline is reference

*SPPB* Short Physical Performance Battery, *TUG* Timed-Up and Go Test, *BI* Barthel Index

^a^Significant time effect compared with baseline

^b^Significant group effect compared with control group

^c^Significant time X group interaction effect

In addition, time was a significant effect factor on SPPB (*p* < 0.001) and TUG (*p* = 0.003), and group was a significant effect factor on 6-Meter Walk test (*p* = 0.049).

After adjusting for age and gender (Table 4), interaction between time and group remained a significant factor for SPPB (B = 2.33, 95% CI: 1.39–3.28, p < 0.001), TUG (B = − 11.15, 95% CI: − 17.23 – − 5.06, p = 0.001) and 6-Meter Walk test (B = 0.17, 95% CI: 0.08–0.25, p < 0.001). Age was a significant effect factor on lower body function (B = − 0.22, 95% CI: − 0.32 – − 0.12, p < 0.001), mobility (B = 0.49, 95% CI: 0.03–0.94, *p* = 0.043), and gait speed (B = − 0.22, 95% CI: − 0.02 – − 0.003, *p* = 0.012).

**Table 4:** Adjusted linear mixed models for SPPB, TUG, 6-Meter Walk test and BI

	Adjusted Fixed Effects^**a**^								
	Time			Group			Time X Group		
	B	95% CI	***p value***	B	95% CI	***p value***	B	95% CI	***p value***
SPPB	−1.27	−1.95 – − 0.58	< 0.001*	− 0.16	− 1.49 – 1.18	0.822	2.33	1.39–3.28	< 0.001**
TUG	7.21	2.71–11.61	0.003*	0.90	−5.42 – 7.22	0.784	−11.15	− 17.23 – − 5.06	0.001**
6-Meter Walk test	−0.03	− 0.10 – 0.03	0.268	0.11	−0.06 – 0.16	0.389	0.17	0.08–0.25	< 0.001**
BI	−0.17	− 4.77 – 4.52	0.942	4.11	−3.91 – 12.13	0.327	0.52	−5.65 – 6.65	0.870

*a* Adjusted for age and gender

Group: control group is reference. Time: baseline is reference

*SPPB* Short Physical Performance Battery, *TUG* Timed-Up and Go Test, *BI* Barthel Index

*Significant time effect compared with baseline

**Significant time X group interaction effect

No differences between groups or assessment moments were found in the individuals’ ability to independently perform ADL even after adjusting models for age and gender (Tables 3 and 4). Although age was a significant effect factor (B = − 0.69, 95% CI: − 1.28 – − 0.11, *p* = 0.026), both CG and EG have increased BI total score from baseline.

## Discussion

The results of this study showed that a 6-month multicomponent exercise intervention improves functional capacity, namely, lower body function, mobility, and gait speed of older adults living with NCD. However, these results did not translate in significant changes in the participants’ capacity to perform ADL independently, regardless of being in the experimental or control group.

Exercise has been recognized as an effective non-pharmacological therapeutic approach for individuals diagnosed with major NCD by slowing disease progression and/or improving their physical health and psychosocial well-being [46]. Several meta-analytic and systematic review studies highlighted the beneficial effects of various modalities of exercise (mainly aerobic and strength) on physical fitness and functional outcomes like gait speed, walking endurance, balance, muscle strength, cardiorespiratory fitness, and agility [46, 47]. The present study adds to the already existing literature, by providing evidence on the effectiveness of MT methodology on the functional capacity of individuals living with NCD, namely in the SPPB, TUG, and 6-Meter Walk test, which are greatly associated with individuals’ frailty status, physical disability, and dependency [37, 39, 48].

Our results showed a significant interaction between group (experimental and control) and time (baseline and post-intervention) on SPPB and, more importantly, when analyzing the scores from baseline to post-intervention, a 1-point change (mean difference) was observed for individuals in the EG and CG, which is considered by Know et al. (2009) [35] as a substantial clinically meaningful change. Additionally, the baseline score of SPPB on both groups was on average below 10 points, which is associated with disability and all-cause mortality [37, 49]. After the MT intervention, the score of SPPB increased in EG, resulting in a reduction of this risk comparing to the CG. As stated by Pavasini et al. (2016) [37], in clinical practice settings it is important to consider the SPPB as a prognostic information tool and as a valid outcome to measure intervention effectiveness in people with NCD. In addition, as recently outlined by Borges-Machado et al. (2021) [27], lower body function has been identified as a health-related physical indicator associated with self-rated quality of life in people living with NCD. In contrast to our results, Pitkala et al. (2013) [50] found that both exercise groups (home or group-based) have deteriorated on SPPB after 6-months; moreover, after 12-months of exercise, both groups have significantly declined (2-points) on SPPB. Similarly to our intervention, Barreto and colleagues [24] conducted a 6-month MT exercise program (60-min sessions, twice a week) with individuals diagnosed with dementia living in nursing homes but, in contrast to our study findings, no statistically significant differences were found on lower body function. However, compared to our sample, their participants presented substantially lower baseline scores on SPPB. To the best of our knowledge, the current study is one of the first to consider the effects of a MT intervention on SPPB for individuals with NCD. Further research is needed on this topic.

As a composite measure of functional mobility, TUG requires the executive function (to listen and initiate movements), transfer tasks (to stand and sit down), walking and balance of individuals [41]. Results from our study showed the MT exercise intervention had a significant positive impact in improving the functional mobility of people with NCD, even after adjusting for confounders such as age and gender. Several prospective studies verified that TUG predicted fall risks and nursing home admission; more recently a study with 39.519 individuals conducted by Lee and collaborators (2020) confirmed, after performing follow-up evaluations on a cohort of older adults for approximately 5.7 years, that impaired mobility (TUG > 10 s) was associated with a higher risk of subsequent functional dependency occurrence (e.g., institutionalization or admission to a long-term care facility). Our post-intervention results demonstrate a significant improvement in the EG, which presented a mean score of 11 s on TUG. Conversely, the CG not only presented a higher mean score on TUG (indicative of worsening), as it also shown an incremental score from baseline to post-intervention of 7.2 s, which is superior to the minimal detectable change value of 4.09 s defined by Ries et al. (2009) [42]. Therefore, results from TUG analyzes supports the effectiveness of this intervention by improving the mobility of individuals with NCD, or at least, by minimizing its decline. Similar results were reported by other authors [51–53]. Lam et al.’s (2018) [14] systematic review study with meta-analysis of randomized trials, regarding the effects of exercise on physical functioning and quality of life in individuals with minor and major NCD, revealed strong evidence supporting the exercise in improving TUG and walking speed.

Despite the high gait variability among NCD severity and subtypes (e.g., stride-to-stride fluctuations in distance and time), gait impairment has been associated with neurodegeneration and cognitive impairment, and it is considered a sensitive marker of neurological dysfunction [54, 55]. As highlighted by Cohen & Verghese (2019) [13] abnormal gait is common in major NCD and other neurodegenerative diseases. Designated as the “sixth vital sign” [56], gait assessment must be considered as a crucial part of managing disease, since with major NCD progression, the decline of walking and other motor abilities may lead to loss of mobility and bedridden [13]. Post-intervention results showed that the CG experienced a decline of gait speed, as opposite to the EG that have showed a mean increase of 0.13 m/s, which is a clinically meaningful change [57]. Lam et al. (2018) [14] analyzed seven trials that have evaluated the effect of exercise on gait speed, with 568 individuals diagnosed with moderate-to-severe major NCD, and revealed that exercise improved walking speed by 0.14 m/s. This study results must be highlighted since, even in more advanced stages of the disease, exercise seems effective in improving gait speed. More importantly, the trials that reported significant exercise effects on walking speed adopted MT training methodologies. Finally, the ability to ambulate independently is a major factor contributing to the well-being and autonomy of older populations [13] and, for this reason, must be comprehensively assessed (e.g., clinical history, observation of walking patterns, clinical and neurologic examination) in clinical practice settings.

As highlighted by a recently published systematic review and meta-analysis study [58], low scores on SPPB and gait speed are predictive of both ADL and/or instrumental ADL decline, while the low scores on TUG are associated with worsening ADL among older adults. However, despite the positive effect of our intervention on the functional capacity of individuals with NCD, no significant differences were found in the ability to independently perform ADL, like previous studies [24, 59, 60]. Baseline least square mean scores on BI superior to 80 points on both groups, might be considered as one of the hypotheses that could partially justify the absence of significative differences, since a total score between 60 and 89 points is indicative of being “slightly dependent” [45].

According to Nuzum et al. (2020) [61], it is necessary to consider the role of cognition when analyzing functional independence outcomes, since cognitive deterioration tends to precede and predict functional decline. Therefore, ADL performance might not be linearly correlated with functional capacity in individuals with NCD, as other factors may have a more decisive role in this relationship. Although no baseline differences were found for cognitive performance (measured with MMSE and ADAS-Cog) between groups, authors consider that NCD subtype and/or stage might have influenced individuals’ functional independence. Nevertheless, these analyzes cannot be conducted due to insufficient data on disease severity. Also, although declines in executive functions have been associated with increased dependency on everyday function [15], such functions were not measured in detail.

Clemmensen et al. (2020) [15] in a cross-sectional study concerning the association between physical performance, cognition, and ADL functions, found out a moderate correlation between general cognitive function and instrumental ADL – particularly, processing speed and attention –, as opposite to basic ADL, whereas no correlation was found, suggesting that basic ADL is less cognitively demanding and, therefore, less dependent of cognitive function. Regarding physical performance, these authors’ findings indicated that aerobic fitness, mobility, strength, and endurance of lower extremities were not associated with ADL independence in people with mild-to-moderate Alzheimer’s disease.

Toots et al. (2016) [60] cluster-randomized controlled trial study implemented a 4-month high-intensity functional exercise program with balance and lower body strength exercises. After 4-months of intervention, and at 7-months follow-up evaluation, this study results were consistent with ours, and no significant between-group effects were found in ADL independence. Nevertheless, in interaction analyses these authors found that the effect of exercise was significant in favor of participants with non-Alzheimer’s disease dementia subtype (when comparing those with Alzheimer’s disease) at 7-months in BI scores. Moreover, Toots and colleagues (2016) reinforced that the declines in ADL independence are multifactorial, and improvements in several physical function components may not be reflected in some activities, such as bladder control or feeding.

In addition, Clemmensen and colleagues (2020) also mentioned other factors beyond cognitive functioning, that may also affect individuals’ ability to perform ADL; they are related with the environment inhabited (e.g., physical factors such as lightning and spatial layout) by the person diagnosed with major NCD. Lastly, the living context (e.g., home-dwelling vs. institutionalized) and the availability of caregivers to provide greater opportunities to the care receiver to remain functional and engaged in meaningful activities [61] may also be considered as important factors.

Despite the increasing evidence acknowledging the beneficial effect of exercise on ADL performance, results are still broadly heterogeneous and of low quality [14, 47, 61]. The dose-dependent relationship remains unclear and although the BI has been used globally [62], empirical evidence regarding its validity for assessing individuals with major NCD is still scarce [63]. Even though our previous research demonstrated the beneficial effect of MT intervention in ADL functionality in older adults with major NCD [25], further controlled trials are needed with larger samples considering this training methodology, and the different types/stages of major NCD.

As declared by Cadore et al. (2019) [64], MT exercise is effective in improving most, if not all, of the frailty syndrome hallmarks; for this reason, it may be considered as a cornerstone for frail individuals with cognitive impairment to mitigate/delay and/or improve physical status and cognitive function. Also, MT exercise interventions have been recently recommended by the *World Health Organization* [20] to older populations, and by the *American College of Sports Medicine* [11] specifically to people with Alzheimer’s disease, since targeting multiple modes of exercise might be the most effective training methodology for enhancing flexibility, balance, strength, and endurance.

### Limitations

There are some limitations to the present study that must be acknowledged. First, this study was a non-randomized controlled trial and therefore we cannot exclude the influence of bias estimates on treatment effects. Nevertheless, it is important to highlight that there were no statistically significant differences between the groups at baseline, except for age and number of comorbidities; moreover, participants were allocated according to their availability and not by a researcher’s decision. Second, the study comprised a small sample size, so results should be interpreted carefully and not generalized. Third, the high variability in the NCD diagnosis (stage and subtype) could potentially influence our results; additionally, the authors acknowledge the lack of data on cognitive function and daily physical activity, which could also potentially influence the results. Four, data must be interpreted with caution considering the individuals’ high levels of independence (i.e., with ability to move autonomously without assistive devices and with a relatively high baseline scores on ADL performance measure), which could not totally reflect the characteristics of this specific population. Fifth, a significant number of participants experienced falls (at least one) during the previous 12-months to baseline evaluations, which was not taken into consideration during statistical analysis. Finally, only CG participants received the health-related information sessions.

## Conclusion

Although the dose-dependent relationship remains unclear for each type/stage of major NCD and target outcome, increasing evidence advocates for MT interventions, since the associated physical and mental health benefits overcome eventual related adverse events/detrimental effects [16, 21]. As stated by Nuzum et al. (2020) [61], further research is needed to clarify which training methodology may have a greater impact on individuals’ physical functioning and functional independence in all stages of various major NCD subtypes, considering different age groups and living environment (e.g., institutionalized vs. domiciliary). A MT methodology seems to be an effective strategy to improve individuals’ functional capacity, but further studies are needed to verify its’ impact on daily functionality.

## Data Availability

The dataset supporting the conclusions of this article will be available upon reasonable request addressed to the corresponding author.

## Abbreviations

ADAS-Cog: Alzheimer’s Disease Assessment Scale - Cognitive
ADL: Activities of Daily Living
BI: Barthel Index
CDR: Clinical Dementia Rating
CG: Control Group
DSM-5: Diagnostic and Statistical Manual of Mental Disorders Fifth Edition
DSM-IV-TR: Diagnostic and Statistical Manual of Mental Disorders Fourth Edition – Text Revision
EG: Experimental Group
HRmax: Maximum Heart Rate
ICD-10: International Statistical Classification of Diseases and Related Health Problems-10th Revision
MMSE: Mini Mental State Examination
MT: Multicomponent Training
NCD: Neurocognitive Disorder
NINCDS-ADRDA: National Institute of Neurological and Communicative Disorders and Stroke Alzheimer’s Disease and Related Disorders Association
SD: Standard Deviation
SPPB: Short Physical Performance Battery
TUG: Timed Up and Got Test
